# Evaluating ChatGPT-4o as a decision support tool in multidisciplinary sarcoma tumor boards: heterogeneous performance across various specialties

**DOI:** 10.3389/fonc.2024.1526288

**Published:** 2025-01-17

**Authors:** Tekoshin Ammo, Vincent G. J. Guillaume, Ulf Krister Hofmann, Norma M. Ulmer, Nina Buenting, Florian Laenger, Justus P. Beier, Tim Leypold

**Affiliations:** ^1^ Department of Plastic Surgery, Hand and Reconstructive Surgery, University Hospital Rheinisch-Westfälische Technische Hochschule (RWTH) Aachen, Aachen, Germany; ^2^ Department of Orthopedics, Trauma and Reconstructive Surgery, Division of Arthroplasty, University Hospital Rheinisch-Westfälische Technische Hochschule (RWTH) Aachen, Aachen, Germany; ^3^ Department of Radiation Oncology, University Hospital Rheinisch-Westfälische Technische Hochschule (RWTH) Aachen, Aachen, Germany; ^4^ Department of Diagnostic and Interventional Radiology, University Hospital Rheinisch-Westfälische Technische Hochschule (RWTH) Aachen, Aachen, Germany; ^5^ Institute of Pathology, University Hospital Rheinisch-Westfälische Technische Hochschule (RWTH) Aachen, Aachen, Germany

**Keywords:** sarcoma, multidisciplinary sarcoma tumor board, artificial intelligence, chat-GPT, large language models, cancer, LLM

## Abstract

**Background and objectives:**

Since the launch of ChatGPT in 2023, large language models have attracted substantial interest to be deployed in the health care sector. This study evaluates the performance of ChatGPT-4o as a support tool for decision-making in multidisciplinary sarcoma tumor boards.

**Methods:**

We created five sarcoma patient cases mimicking real-world scenarios and prompted ChatGPT-4o to issue tumor board decisions. These recommendations were independently assessed by a multidisciplinary panel, consisting of an orthopedic surgeon, plastic surgeon, radiation oncologist, radiologist, and pathologist. Assessments were graded on a Likert scale from 1 (completely disagree) to 5 (completely agree) across five categories: understanding, therapy/diagnostic recommendation, aftercare recommendation, summarization, and support tool effectiveness.

**Results:**

The mean score for ChatGPT-4o performance was 3.76, indicating moderate effectiveness. Surgical specialties received the highest score, with a mean score of 4.48, while diagnostic specialties (radiology/pathology) performed considerably better than the radiation oncology specialty, which performed poorly.

**Conclusions:**

This study provides initial insights into the use of prompt-engineered large language models as decision support tools in sarcoma tumor boards. ChatGPT-4o recommendations regarding surgical specialties performed best while ChatGPT-4o struggled to give valuable advice in the other tested specialties. Clinicians should understand both the advantages and limitations of this technology for effective integration into clinical practice.

## Introduction

Soft tissue sarcomas (STS) are a rare form of tumors, affecting approximately less than 1% of the population (1). Due to their heterogeneous nature, the management of STS requires a multidisciplinary approach, making tumor boards crucial for optimal decision-making (2–4). The complexity and variability of STS cases demand input from various specialists, including oncologic surgeons, plastic surgeons, radiation therapists, radiologists, and pathologists.

The use of artificial intelligence (AI) in healthcare is gaining increasing interest and is expected to drastically change the way how patients will be treated (5). Large Language Models (LLM) are a type of AI designed to understand and generate human language. These models are trained on extended databases, enabling them to learn the patterns, grammar, and structure of language. They can then use this knowledge to perform a wide range of tasks, such as answering questions, translating languages, summarizing text, generating content, and engaging in conversations. Among the various LLM, ChatGPT in particular, which was launched in March 2023, has attracted significant attention because of its understanding of natural language and its wide range of applications, including the health care system (6). Incorporating AI in daily practice could aid clinicians in decision making in complex and time-consuming cases.

The use of AI in a tumor board has been explored in only five studies so far, which covered selected cancer entities, i.e. breast cancer, gastric cancer, head and neck cancer and as an adjunct in decision making in molecular tumor boards (7–11).

Sorin et al. assessed ChatGPT-3.5 as a decision support tool for breast tumor boards (8). They entered clinical data from ten patients and compared the model’s recommendations to those of the tumor board. In 70% of cases, ChatGPT’s suggestions aligned with the board’s decisions. Two radiologists graded the chatbot’s performance in summarization, recommendations, and explanations, with scores generally indicating moderate to high agreement. The study suggests that LLM could assist in clinical decision-making but highlights the need for clinicians to understand both the benefits and limitations of such technology.

Furthermore, Griewing et al. evaluated ChatGPT-3.5 treatment recommendations against those of a multidisciplinary breast cancer tumor board (12). The overall concordance was 50%, increasing to 58.8% for invasive breast cancer cases. They concluded that due to occasional inaccuracies, current LLM technology is not yet fully reliable as a support tool for tumor boards.

These previous studies revealed first promising results of ChatGPT in the context of a multidisciplinary tumor board (MTB), yet these studies also demonstrated that LLM are not yet sufficiently advanced for reliable clinical application.

To reach the full potential of LLM, “prompt-engineering” can be used as a tool to improve the performance of Chat-GPT (13).

In previous studies, we analyzed how ChatGPT-4 can assess complex clinical scenarios, suggest viable diagnostic treatment options, and effectively take comorbidities into account using a range of prompting techniques (14–16). We demonstrated that prompt-engineering was able to improve the function of LLM when asked about reconstructive procedures of the upper extremity (14). As a consultation assistant in a hand surgery outpatient clinic, the function of ChatGPT-4 was likewise enhanced by prompt-engineering techniques (15). However, further research is required to explore its reliability and practicality in actual practice.

So far, however, there have been no studies showing the effectiveness of ChatGPT or other LLM in a multidisciplinary sarcoma tumor board (MSTB). Additionally, previous studies on the performance of Chat-GPT on multidisciplinary tumor boards did not focus on the usage of prompt-engineering. Thus, we tested OpenAI’s most advanced version of GPT-4o at the time of conducting this study, with five complex cases of sarcoma mimicking real-world cases and analyzed its answers with experienced physicians who regularly participate in real tumor boards at our university hospital.

## Materials and methods

In this study, ChatGPT-4o was confronted with five simulated clinical cases representing various types of sarcomas, carefully selected to reflect real-world clinical scenarios and its answers were recorded and analyzed. The cases included: (1) myxoid liposarcoma, (2) malignant peripheral nerve sheath tumor, (3) desmoid tumor, (4) dermatofibrosarcoma protuberans, and (5) dedifferentiated liposarcoma. Given that liposarcomas are a relatively common subtype of soft tissue sarcomas, while malignant peripheral nerve sheath tumors are a rare and highly aggressive form this selection was designed to cover a broad spectrum of sarcoma entities (17). Malignant peripheral nerve sheath tumors, for example, account for approximately 5-10% of all soft tissue sarcomas. They are characterized by a high clinical aggressiveness and complexity (18). Additionally, dermatofibrosarcoma protuberans constitutes about 1% of soft tissue tumors, although they are not classified as soft-tissue sarcomas, approximately 10-15% can transform into sarcomatous lesions (19). The purpose to include both high-risk as well as rare and more commonly encountered mesenchymal tumors was to test GPT-4o more comprehensively.

Notably, we utilized the latest version of the model, GPT-4o, launched in May 2024, which was designed to offer improved speed and accuracy compared to earlier versions (6). Current studies showed a better performance of its predecessor GPT-4 in comparison to the earlier model GPT-3.5 (20, 21).

To improve the effectiveness in the specialized setting of a MSTB, specific prompt-engineering methods were employed to prime GPT-4o.

Initially, we used the technique of “role prompting”, assigning GPT-4o to the specific role of assisting in a MSTB and clearly defining the context for GPT-4o.

We explained the standard diagnostic algorithm for sarcoma care as laid out in the German guidelines for sarcoma care to GPT-4o to ensure that it adheres to it (22):

1. Imaging: MRI as the gold standard, CT, X- Ray and Sonography
2. Biopsy: punch biopsy, excision biopsy (tumor <3cm), incision biopsy
3. Multidisciplinary/interdisciplinary evaluation
4. Surgical resection with the pathological examination of the mass after resection
5. Radiotherapy
6. Chemotherapy
7. Aftercare
8. Rehabilitation

Next, we defined the role of each specialty, which participated in this study. This technique is described as “expertise emulation”. For example, to ensure that it comprehends the role of a plastic surgeon, we submitted the phrase “As a Plastic Surgeon you should create a plan to reconstruct a possible defect. Suggest specific free flaps, nerve transfers or tendon transfers for reconstruction. Please provide concise but comprehensive explanations of each technique, including their applications, preoperative planning, risks, limitations, and expected results. Begin with the most appropriate method.”

Chain of Thought (CoT)-prompting is a method of enhancing the precision of GPT-4o final decisions by prompting LLM articulate and elucidate their reasoning process. It allows LLM to break down multi-step problems into smaller, more manageable steps. Additionally, it can be applied to various tasks, such as medical decision making, diagnosis and reasoning through complex clinical scenarios, much like the multi-step reasoning used by human experts in tumor boards (23).

Zero-shot prompt engineering is a technique where prompts are used to LLM without including any specific examples or prior cases in the prompt itself (24). In this approach, the model is asked to perform a task or provide information based purely on its general training and pre-existing knowledge. Accordingly, we implemented the “zero-shot chain-of-thought-method” initially described by Kojima et al. (25), who observed the enhancement of the LLM performance by triggering a thought process by introducing the phrase “Let’s think step by step” devoid of any particular examples. Moreover, we told GPT-4o to “provide a step-by-step treatment recommendation with regard to the necessity of a biopsy, present or future surgical treatment, systemic treatment and radiation therapy taking the given patient information into consideration.”

To conclude the prompting process, GPT-4o was instructed to “sum up the recommendations and create an overall tumor board decision for the patient”. Once GPT-4o confirmed its readiness following the established prompts, five simulated cases were submitted to GPT-4o.

For each case, GPT-4o was given detailed patient information with a comprehensive patient history. The model was then tasked with summarizing the case and providing recommendations for each specialty involved in the treatment of sarcoma. Finally, GPT-4o had to compile a comprehensive summary of its recommendations.

These cases, along with GPT-4o’s recommendations, were then presented to a panel of five experienced and specialized sarcoma experts, including an oncologic surgeon, a plastic surgeon, a radiation therapist, a radiologist, and a pathologist. Each expert, holding board certifications of its specific specialty, independently carried out the evaluation process. They rated the chatbot’s answers based on their accuracy and usefulness in clinical decision-making on a Likert scale based on five different criteria. Each criterion was scored on a scale of 1 (I completely disagree) to 5 (I completely agree) (**Table 1**). Every response produced by GPT-4o underwent rigorous assessment by the expert panel focusing on five specific criteria.

**Table 1:** Five different Criterions rated on a Likert Scale from one to five, one being the lowest and five being the highest score.

1	The AI fully understood the case.
2	I agree with the AI regarding the therapy/diagnostics in my area of expertise.
3	The AI provides useful recommendations for follow-up care and rehabilitation.
4	The AI summarizes the cases well and formulates a sensible tumor board decision.
5	The AI could be a valuable addition to the tumor board.

Statistical analysis was computed with GraphPad Prism 10 (GraphPad Software Inc., USA) and all data are presented as mean ± standard deviation. Data distribution was tested by Shapiro-Wilk-normality test. In normally distributed data one-way ANOVA followed by Tukey’s *post-hoc* test was performed. For non-normally distributed data, the Kruskal-Wallis H-test was used, followed by Dunn’s *post-hoc* correction. Statistical significance was defined as p≤ 0.05.

## Results

The complete chat history with GPT-4o can be viewed via the links in **Table 2**.

**Table 2:** Five simulated cases and ChatGPT-4o’s response.

Case	ChatGPT-4o
1	https://chatgpt.com/share/901b1b21-ca9e-4995-a82b-beb86d4f305b
2	https://chatgpt.com/share/d5ea2cda-d571-4ee6-890c-c1b05f96c067
3	https://chatgpt.com/share/e40b100a-b788-490a-9c5b-3a6f3832b2fa
4	https://chatgpt.com/share/1114a9a3-abb6-4503-b44f-535bb614f9b2
5	https://chatgpt.com/share/cd53af57-8844-46c2-bced-19ab9010fcb7

**Figure 1:**
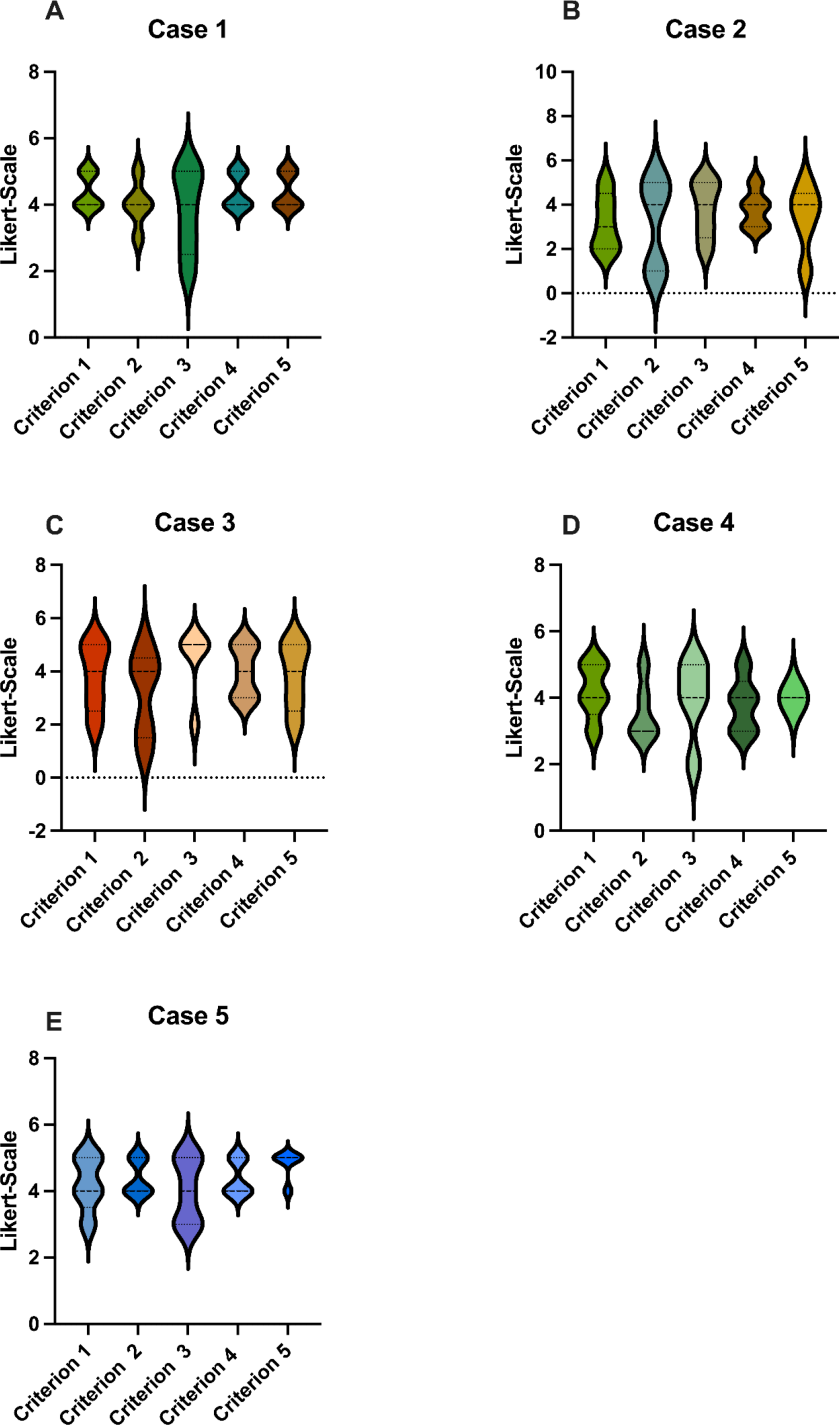
Scores: GPT-4o’s responses were evaluated using a Likert Scale based on the five criteria **(A-E)**, as seen in **Table 1**. These graphs show the average score for each case, where 5 is the highest possible score and 1 is the lowest. GPT-4o, Generative Pretrained Transformer 4o.

As a part of the analysis for Criterion 1, “The AI fully understood the case”, GPT-4o received a cumulative score of 19.6, attaining an average rating of 3.92 (see **Figure 1**). The evaluators therefore agreed that GPT-4o understood the cases considerably well.

For Criterion 2, “I agree with the AI regarding the therapy/diagnostics in my area of expertise”, GPT-4o achieved a cumulative score of 18, with an average rating of 3.6, which corresponds to a moderate score (see **Figure 1**). Notably, the radiologist and pathologist did not agree with the diagnostic recommendations of GPT-4o.

Regarding Criterion 3, “The AI provides useful recommendations for follow-up care and rehabilitation”, GPT-4o received a cumulative score of 19.8, attaining an average rating of 3.96 with a similar score as Criterion 1 (**Figure 1**). This score demonstrated the ability of GPT-4o to create useful follow-up and rehabilitation recommendations, in which GPT-4o performed moderately good.

Assessing Criterion 4, “The AI summarizes the cases well and formulates a sensible tumor board decision”, GPT-4o obtained a cumulative score of 20, with an average rating of 4 (**Figure 1**). The evaluators therefore agreed that GPT-4o was able to formulate a sensible tumor board decision.

In consideration of Criterion 5, “The AI could be a valuable addition to the tumor board”, GPT-4o achieved a cumulative score of 19.4, reaching an average rating of 3.88 (**Figure 1**). One of the evaluators expressed that GPT-4o would not be a valuable addition to the tumor board in the future whereas four of the evaluators held a contrary view.

**Figure 2:**
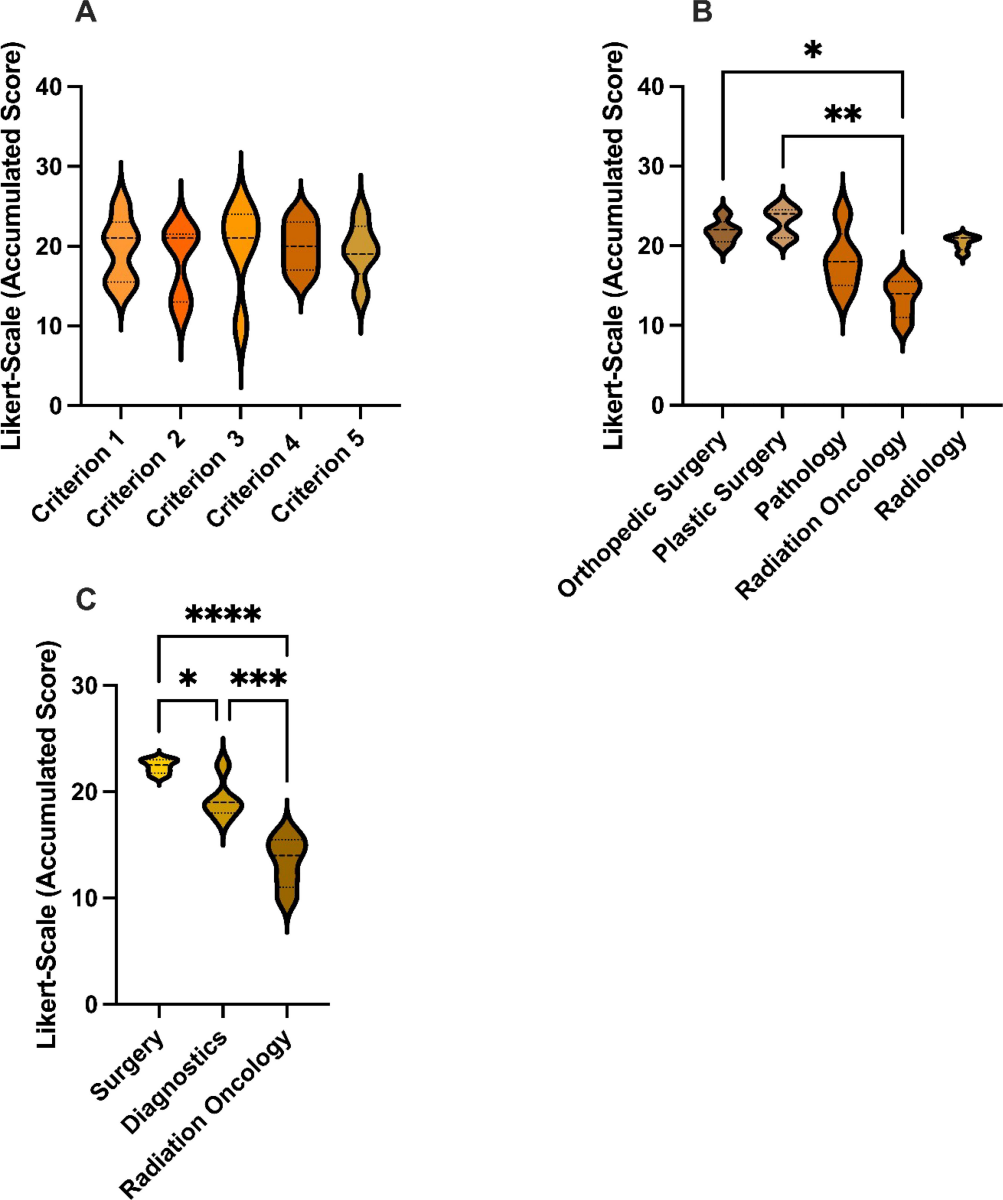
Accumulated scores: **(A)** Accumulated scores for GPT-4o’s responses for each Criterion. **(B)** Accumulated scores for GPT-4o’s responses for each specialty. **(C)** Accumulated scores for GPT-4o’s responses for each specialty group. This graph shows the average score for each case, where 25 is the highest possible score and 5 is the lowest. GPT-4, Generative Pretrained Transformer 4. *p < 0.05, **p < 0.01, ***p < 0.001, ****p < 0.0001.

There were no statistical differences between the five criterions (see **Figure 2A**). Likewise, the individual cases did not differ significantly (**Figure 1**). GPT-4o achieved an overall average score of 3.76 across all criterions, corresponding to a cumulative average score of 18.8, reaching 75.2% of the maximum score. This reflects a uniformly moderate effectiveness of the AI across different cases, consistent with our previous study that also found no differences among cases with varying difficulty (14–16).

Matching specialties were grouped into Surgery (SUR), Diagnostics (DIA), and Radiation Oncology (RAD) and subgroup analysis was performed (**Figure 2**). Of the three groups, the surgical specialties ranked GPT-4o’s performance best with a mean score of 4.48, which was statistically significantly higher than the diagnostic specialties and radiation oncology (p< 0.05). The Diagnostics group exhibited a mean score of 3,86 and outperformed (p< 0.05) the radiation oncology with an average score of 2.68.

**Figure 3:**
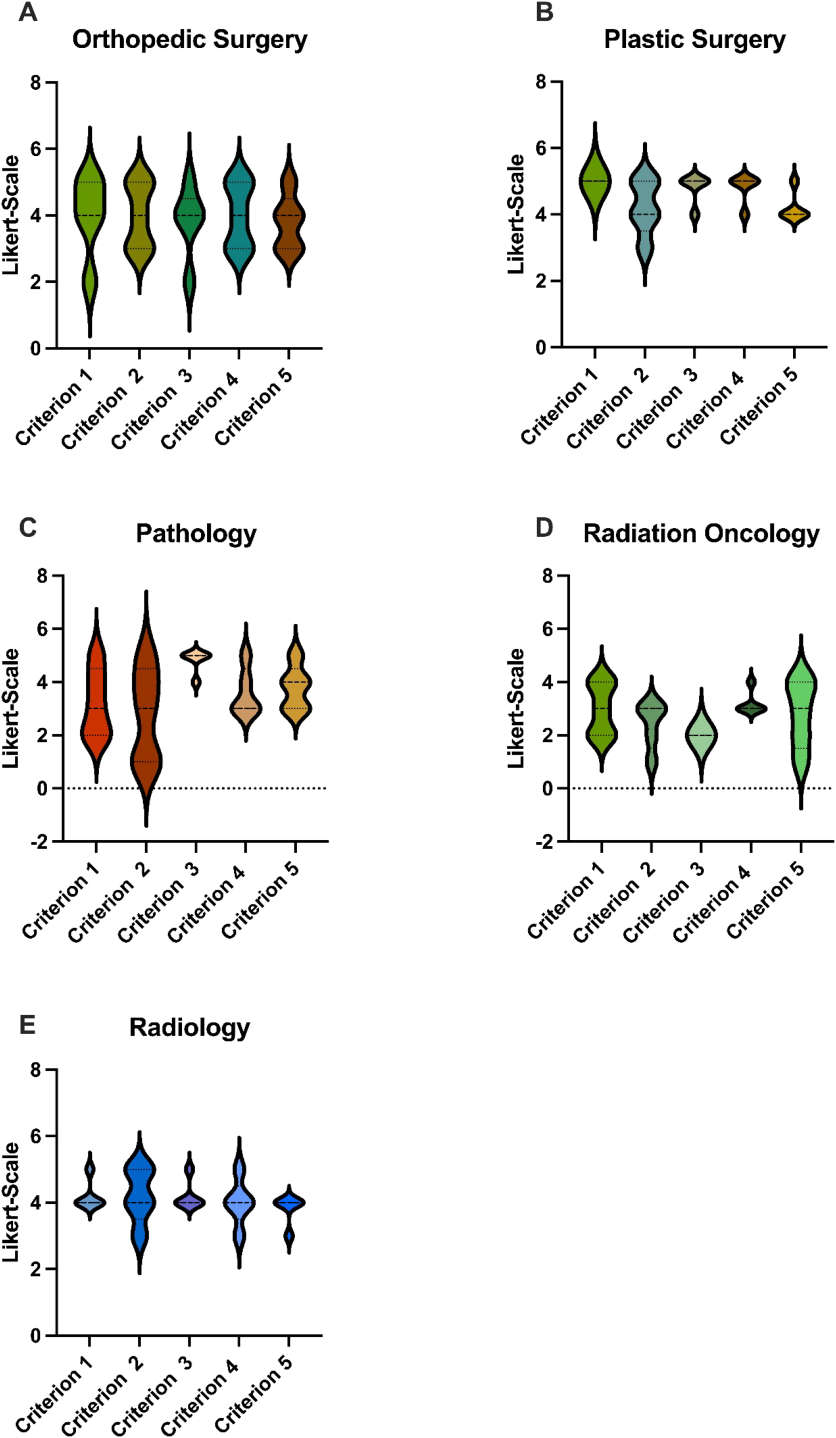
Scores: GPT-4o’s responses were evaluated using a Likert Scale based on the five criteria. These graphs show the average score for each specialty in terms of the five different cases, where 5 is the highest possible score and 1 is the lowest. **(A)** Accumulated Scores by the Orthopedic Surgeon for GPT-4o’s responses for each Criterion, **(B)** Accumulated Scores by the Plastic Surgeon for GPT-4o’s responses for each Criterion, **(C)** Accumulated Scores by the Pathologist for GPT-4o’s responses for each Criterion, **(D)** Accumulated Scores by the Radiaton Oncologist for GPT-4o’s responses for each Criterion, **(E)** Accumulated Scores by the Radiologist for GPT-4o’s responses for each Criterion GPT-4o, Generative Pretrained Transformer 4o. p < 0.05.

Single specialty analysis (see **Figure 3**) likewise showed that orthopedic- and plastic surgery had ranked significantly higher than radiation oncology. There were no statistical differences between the other specialties.

## Discussion

Since its launch, ChatGPT and LLM in general sparked ample interest within the research community on their usability in the medical sector due to their unique features and unprecedented performance. However, LLM in their current state are regarded as immature and not fully applicable for clinical implication yet (26–29).

Regarding the use of ChatGPT in the context of tumor boards, there are several studies which examine the impact of ChatGPT in particular and LLM in general on multidisciplinary tumor boards (8, 9, 11, 29).

One of these studies evaluated ChatGPT as a support-tool for the multidisciplinary tumor board decision-making process in primary breast cancer cases (30). This study compared ten cases discussed in a multidisciplinary tumor board at their department and presented those cases to ChatGPT-3.5. ChatGPT was able to identify risk factors for hereditary breast cancer and identified an elderly patient with an indication for chemotherapy. They concluded that ChatGPT is capable of formulating conclusive answers on a superficial level. However, it is evident that the model lacks the robust data necessary for effective training.

Further, Sorin et al., conducted a study of ten patients presented in a breast tumor board at their institution and asked ChatGPT-3.5 to recommend therapy options (8). The answers of ChatGPT were compared to the tumor board decisions and were graded by two radiologists. ChatGPT recommendations aligned with tumor board decisions in seven out of ten cases (70%), which is consistent with the results of our study, especially with regard to the rating by our radiologists. We reached a slightly better performance with 75.2% which may be due to the usage of established prompt-engineering techniques. These results support our conclusion about the moderately good effectiveness of LLM as a possible support tool for multidisciplinary tumor boards in their current state. We see various limitations, however, in the study design. While Sorin et al. did not utilize prompt-engineering in their approach, our study employed this technique to refine the model’s responses. Additionally, our cases were evaluated through an interdisciplinary approach, contrasting with Sorin et al. use of singular expertise.

Griewing et al. carried out a concordance assessment by comparing ChatGPT 3.5 treatment recommendations with those of a multidisciplinary breast tumor board (11). Their study reported an overall concordance of 50%, which increased to 58.8% for invasive breast cancer cases.

In our study, GPT-4o achieved 75.2% of the maximum score, which indicates a better performance. However, we used a Likert scale for evaluation, which complicates a direct comparison of results. Griewing et al. also did not employ established prompt-engineering techniques, which may have caused the lower concordance rates observed.

As mentioned above, many studies compared ChatGPT answers and multidisciplinary breast tumor boards, showing moderate to moderately good performance by ChatGPT.

To investigate the impact of different LLM on head and neck cancer cases Schmidl et al. (9) challenged ChatGPT-4 and Claude-3-Opus with 50 head and neck cancer patients discussed in their multidisciplinary tumor board. Claude-3-Opus outperformed ChatGPT-4 with regard to the correct diagnostic workup of patients. In terms of clinical recommendations, the authors found that in terms of explanation and summarization, Claude 3 reached similar scores to ChatGPT-4.0. Nonetheless, the authors stated that advanced AI models like ChatGPT4 and Claude-3-Opus can be merely used as an adjunct in MTB given their occasional wrong recommendations. One advantage of that study is the direct comparison of two LLM. However, the prompt utilized by those authors does not align with established prompt-engineering techniques but rather resembled case presentations (22–24).

Medical tumor boards typically discuss complex clinical cancer cases. The active involvement of oncologists, surgeons, radiologists, radiation oncologists, and pathologists in tumor boards at the same time is labor-intensive and requires expert knowledge, which limits the usage of tumor boards in most countries to tertiary referral centers or similar structures worldwide. Therefore, it would be highly beneficial if the expertise and knowledge of sarcoma tumor boards were not restricted solely to those highly specialized referral centers. Low-income and developing countries as well as rural areas could benefit from the availability of easily integrable software which could replace the expert panel of a tumor board. Given that sarcomas are rare and complex tumors, access to specialized tumor boards is crucial for accurate diagnosis and treatment planning (1–4). Integrating ChatGPT in the field of oncology could prove valuable for cancer research, diagnostics and patient support (7). As the medical sector worldwide faces a specialized worker shortage, the deployment of LLM in general could alleviate some of these constraints.

There are only a few studies that have investigated the performance of ChatGPT or artificial intelligence regarding Sarcoma cases or Sarcoma related questions. Mastuoka et al. evaluated ChatGPT-4’s performance in aligning with the Japanese Orthopaedic Association (JOA) guidelines for soft tissue sarcoma management. ChatGPT achieved an 86% alignment rate, with two cases of complete alignment and 17 partial alignments, but showed discrepancies in specific treatment areas. They concluded that the 14% of nonaligned responses underscore the need for continuous improvement and rigorous validation of AI recommendations to ensure alignment with current medical practices (31).

Another study evaluated the performance of ChatGPT-3.5 and ChatGPT-4 using 80 clinical questions derived from the German S3 guideline for adult soft tissue sarcoma (STS). ChatGPT-4 outperformed ChatGPT-3.5 in accuracy and adequacy, particularly in general STS treatment and extremity/trunk sarcoma questions. However, they concluded that both models occasionally provided misleading or potentially dangerous information, highlighting the need for cautious use and human oversight in clinical settings (32).

Valentini et al. assessed the quality of ChatGPT’s responses to 25 sarcoma-related questions, evaluated by three independent sarcoma experts using five criteria: completeness, misleadingness, accuracy, timeliness, and appropriateness. The median score was 18.3 out of 25. While ChatGPT performed well on general questions and definitions, its performance was weaker on treatment-related inquiries, where only 45% of responses were rated as good or very good. The authors highlighted the inconsistent quality of ChatGPT’s answers for rare diseases like sarcoma and stressed the importance of sarcoma physicians warning patients about the risk of misinformation (33).

To our knowledge, this is the first study evaluating the role of ChatGPT as a support tool for multidisciplinary sarcoma tumor boards. To enhance the capability of GPT-4o, we employed specific prompting techniques as outlined in the methodology.

In this study GPT-4o achieved 75.2% of the maximum score, which indicates a moderate to good performance. The surgical specialties scored significantly better than the diagnostic specialties and radiation oncology, this may be due to the fact that the basic indication for radical tumor resection is always given. Between the pathologist and radiologist, which were grouped into the diagnostic group, the rating was comparable. A possible explanation for the negative assessment by the radiation oncologist could be that, particularly in rare tumors such as sarcomas, extensive discussion is required. Given the heterogeneity of these tumors, standardized treatment approaches are often insufficiently established. Also, no imaging data were provided, which are essential for a radiologist`s assessments. Instead, only radiological features were listed. This absence of imaging limited the ability to fully simulate the diagnostic process and integrate visual information critical for accurate evaluations.

The cumulative score of 3.92 for Criterion 1 indicates the general ability of ChatGPT to understand the cases in the context of all findings, including symptoms, histological and radiological criteria but it lacks the capability to fully comprehend the presented cases.

GPT-4o received the lowest score for Criterion 2. One possible explanation may be the agreement of therapeutic and diagnostic recommendations within the pathologist, radiologist and radiation oncologist, rating this specific criterion significantly lower than the surgical group. This could be explained by the lack of data provided to the AI or the underlying fundamental lack of specific data it was trained on. The surgical excision of a tumor is a more straightforward concept despite the technical challenges than multistep diagnostic and radiation oncology treatment modalities.

Also, our evaluators confirmed the moderate efficacy of ChatGPT to provide useful follow-up and rehabilitation recommendations, which is essential for patient follow-up to detect potential future recurrences of a tumor at an early stage, thereby increasing the likelihood of survival (34).

Criterion 4 received the highest score, which assesses the ability to formulate and to summarize a reasonable tumor board decision. In our view, this is a pivotal criterion as it represents one of the core functions of a tumor board.

Furthermore, in Case 1, ChatGPT recommended only neoadjuvant radiotherapy, despite the criteria for neoadjuvant chemotherapy being fulfilled, including the presence of a high-risk soft tissue sarcoma (STS) characterized by a size >5 cm, deep-seated location, grade 2/3 histology, and being a chemosensitive subtype. The patient would therefore have been under-treated. A limitation in Case 2, which involves a malignant peripheral nerve sheath tumor (MPNST), is the lack of clear specification regarding tumor size. This omission leaves neoadjuvant chemotherapy as a potential preoperative treatment option that cannot be definitively ruled out. To provide more accurate and meaningful therapy recommendations, an AI system would need to proactively identify and request such missing critical information in the future. Case 2 also received a low score of 1 (completely disagree) for Criterion 2 by the Pathologist due to the AI’s misclassification as MPNST. While MPNST typically show weak and focal S100 expression in only 25% of cases, the reported strong and diffuse expression contradicts this diagnosis, except in rare epithelioid forms. Additionally, the interpretation of “TP53 expression” lacked differentiation between wild-type and mutation patterns, further highlighting the AI’s overly simplistic assessment of immunohistology (18, 35).

As for Case 3, according to the consensus of the Desmoid Tumor Working Group, active surveillance is recommended as the first-line therapy for patients with newly diagnosed desmoid tumors. In this case, the AI did not sufficiently emphasize that such tumors should only be surgically treated if they cause functional impairment, such as interference with vital structures or significant physical dysfunction. In Case 5, ChatGPT’s therapeutic recommendation did not include neoadjuvant chemotherapy or radiotherapy, both of which are explicitly recommended by the German STS S3 guidelines.

Our study shows a moderate to good performance of GPT-4o in understanding the presented cases, providing useful follow-up and rehabilitation recommendations and formulating a sensible tumor board decision. We, therefore, conclude that especially with the advancement of prompt-engineering-techniques, LLM could be a valuable and useful tool for assisting in a MSTB in the future despite current limitations. With the rapid advancements in AI development in recent times, it appears reasonable that its capabilities will continue to evolve steadily. As tumor boards increasingly rely on collaborative decision-making, physicians must understand how AI systems operate, along with their potential opportunities and risks. Although AI is unlikely to replace a multidisciplinary tumor board entirely, this study highlights a practical way in which AI in the future could improve the overall treatment process, particularly in the context of multidisciplinary care.

In this study, fictional cases rather than real anonymized patient cases were presented to GPT-4o. Consequently, we were unable to compare our findings with actual previous tumor board decisions. However, this approach was not feasible due to strict data privacy concerns. We also only used ChatGPT-4o and did not compare the answers of different LLM with each other to find out which LLM performs best. At the time this study was conducted, OpenAI’s latest large language model (LLM) available was GPT-4o. By September 2024, the next Version ChatGPT-o1, had been released. Future investigations involving ChatGPT-o1 would be of great interest and could further enhance our understanding of the topic.

One limitation or our study is its monocentric approach with each specialty group being represented by only one evaluator. With a larger group of evaluators from each specialty and inclusion of other centers, the robustness and reliability of the assessments could be significantly enhanced. Another limitation of this study is that in terms of diagnostics no histological slides or radiological imaging were available; instead, only keywords describing the histology or radiological imaging were provided. For an accurate histopathological and radiological diagnosis, a large number of slides alongside a macroscopic examination of the tumor and imaging data would be necessary.

Tumor board decisions are often strictly guided by country-specific clinical guidelines. In this context, the primary strength of a large language model (LLM)—its ability to access and integrate information from a wide range of sources—could become a disadvantage if it leads to deviations from these guidelines. Future studies could explore adapting the prompting strategies to address this limitation. For instance, tailoring prompts to ensure the AI relies exclusively on the relevant country-specific guideline might improve the quality of responses. One potential approach could involve providing the AI with the full guideline as a PDF or structured text prior to case processing.

These constraints could be addressed by future studies, which should be carried out multicentric with multiple evaluators for each specialty. Furthermore, these studies could investigate and compare different LLM to identify the most suitable one.

## Conclusions

This study offers insights into the application of prompt-engineered LLM as decision support tools in sarcoma tumor boards. Our findings suggest that while LLM can provide valuable assistance in setting up a treatment plan, their present limitations, such as variability in performance and inaccuracies, hamper their integration into clinical practice in their current state. Thus, clinicians must understand potential benefits such as improved efficiency and decision-making support as well as the constraints of this technology. Further refinements of LLM in the near future with an improved accuracy and a diminished error rate could pave the way for the integration of LLM into routine clinical practice of tumor boards. However, given the current limitations and error rates, their use poses a potential safety concern and underscores the need for caution when utilizing LLMs like ChatGPT-4o as supportive tools in clinical decision-making.

## Data Availability

The original contributions presented in the study are included in the article/supplementary material. Further inquiries can be directed to the corresponding author.
